# Increasing Physical Activity Amongst Overweight and Obese Cancer Survivors Using an Alexa-Based Intelligent Agent for Patient Coaching: Protocol for the Physical Activity by Technology Help (PATH) Trial

**DOI:** 10.2196/resprot.9096

**Published:** 2018-02-12

**Authors:** Ahmed Hassoon, Jennifer Schrack, Daniel Naiman, Dina Lansey, Yasmin Baig, Vered Stearns, David Celentano, Seth Martin, Lawrence Appel

**Affiliations:** ^1^ Department of Epidemiology Johns Hopkins Bloomberg School of Public Health Johns Hopkins University Baltimore, MD United States; ^2^ Johns Hopkins University School of Medicine Johns Hopkins University Baltimore, MD United States; ^3^ Welch Center for Prevention, Epidemiology, & Clinical Research Johns Hopkins University Baltimore, MD United States; ^4^ Department of Applied Mathematics and Statistics Whiting School of Engineering Johns Hopkins University Baltimore, MD United States

## Abstract

**Background:**

Physical activity has established health benefits, but motivation and adherence remain challenging.

**Objective:**

We designed and launched a three-arm randomized trial to test artificial intelligence technology solutions to increase daily physical activity in cancer survivors.

**Methods:**

A single-center, three-arm randomized clinical trial with an allocation ration of 1:1:1: (A) control, in which participants are provided written materials about the benefits of physical activity; (B) text intervention, where participants receive daily motivation from a fully automated, data-driven algorithmic text message via mobile phone (*Coachtext*); and (C) Voice Assist intervention, where participants are provided with an in-home on demand autonomous Intelligent Agent using data driven Interactive Digital Voice Assist on the Amazon Alexa/Echo (*MyCoach*).

**Results:**

The study runs for 5 weeks: a one-week run-in to establish baseline, followed by 4 weeks of intervention. Data for study outcomes is collected automatically through a wearable sensor, and data are transferred in real-time to the study server. The recruitment goal is 42 participants, 14 in each arm. Electronic health records are used to prescreen candidates, with 39 participants recruited to date.

**Discussion:**

This study aims to investigate the effects of different types of intelligent technology solutions on promoting physical activity in cancer survivors. This innovative approach can easily be expanded and customized to other interventions. Early lessons from our initial participants are helping us develop additional advanced solutions to improve health outcomes.

**Trial Registration:**

Retrospectively registered on July 10, 2017 at ClinicalTrials.gov: NCT03212079; https://clinicaltrials.gov/ct2/show/NCT03212079 (Archived by WebCite at http://www.webcitation.org/6wgvqjTji)

## Introduction

There is consistent evidence that identifies poor dietary choice and physical inactivity as major contributors to death in the US and worldwide [1-3]. Physical activity promotion and adverse health behavior prevention strategies can improve health and reduce subsequent disease for individuals and populations [4]. Despite this evidence, only a fraction of the U.S. population adheres to the recommended guidelines [5]. Behavioral interventions for lifestyle modification (walking) have been successful in research settings [6], but translating complex research interventions, particularly in physical activity, into practice remains problematic [7,8].

Recent advances in hardware technologies, statistical methods, big data processing, and cloud-based computing have resulted in artificial intelligence technologies that may offer efficient, low cost and potentially scalable solutions to deliver individualized behavioral interventions to at-risk populations. Further, the development of intelligent technology solutions via ecological momentary assessment provides unique opportunities for behavioral intervention at the individual level, which may increase adherence and promote long-term lifestyle change after the intervention is completed. To test the utility of such technology, our team constructed artificial intelligent agents (IA) to help cancer survivors become more active throughout their daily routines, via a technology-driven clinical trial designed to deliver affordable, scalable, easy to adopt, individualized behavioral interventions. Currently, the IA approach is being tested in a three-arm randomized trial: The Physical Activity by Technology Help (PATH). The trial compares the two technologies—a voice activated intelligent agent and a more traditional intelligent text messaging—compared to a self-driven traditional behavior change (control). The aim of this pilot study is to assess the preliminary effect of a 4-week intervention by different technological approaches to increase daily physical activity, defined as walking (eg, 10,000 steps per day), among overweight and obese cancer survivors, with a special interest in under-represented African American women, in the state of Maryland. In addition, we developed an innovative approach to identify study candidates from review of Epic Electronic Medical Records (EMR). While this IA is specific to cancer survivors, the methods and technology design may be replicated in the general population.

## Methods

### Design

**Figure 1:**
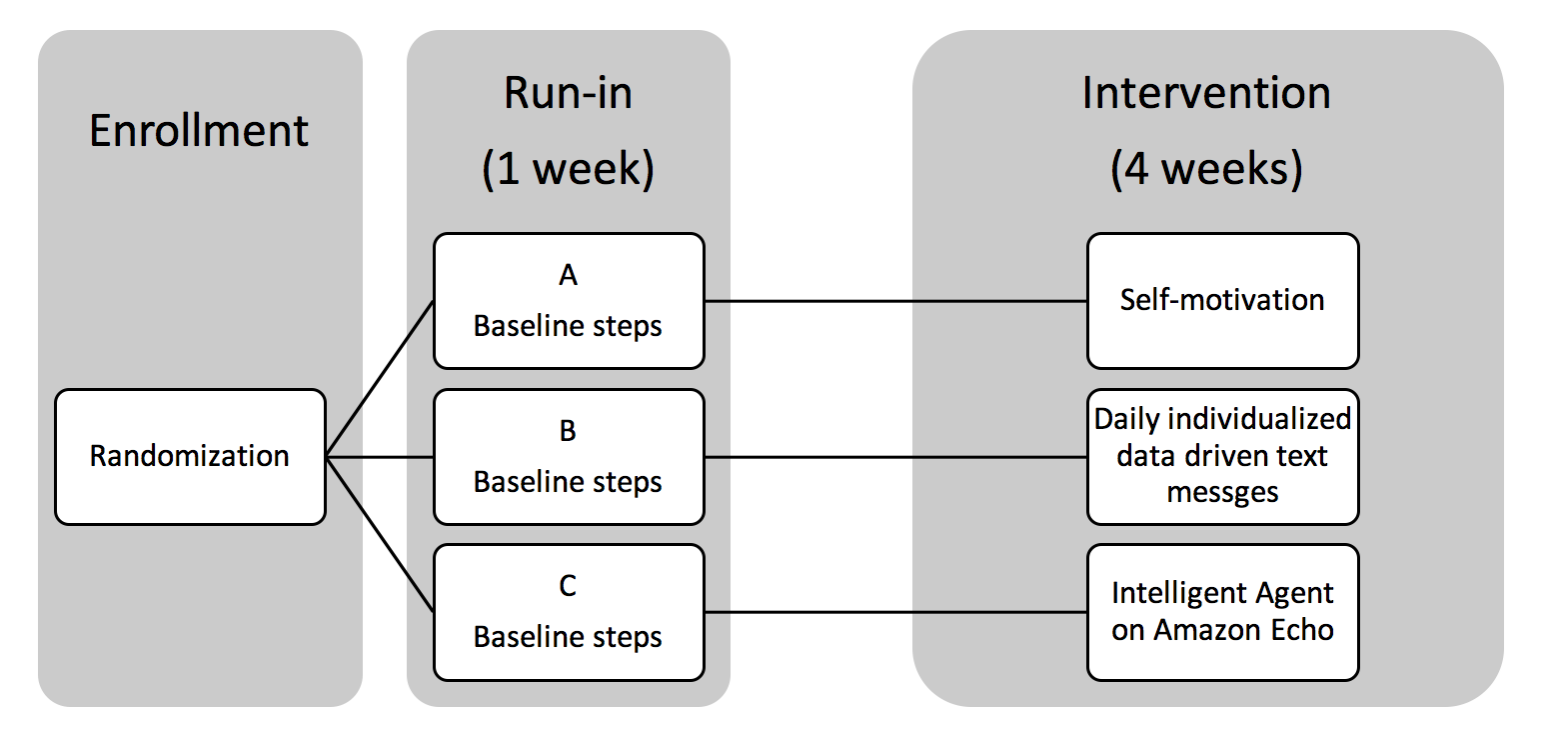
Physical Activity by Technology Help (PATH) study design.

This is a three-arm single center pilot randomized trial with allocation ratio of 1:1:1. The arms are: (A) control, in which participants are provided with written materials about the benefits of physical activity; (B) text-messaging intervention, where participants receive multiple motivational messages from an automated, data-driven algorithmic text message program via mobile phone (*Coachtext*); and (C) voice-assist intervention, in which participants are provided with an in-home autonomous Intelligent Agent using data driven Interactive Digital Voice Assist on the Amazon Alexa/Echo (*MyCoach*).The trial duration is 5 weeks – a one-week run-in phase to establish baseline physical activity, followed by 4 weeks of intervention (Figure 1). Physical activity outcomes are captured via wearable sensors (Fitbit Charge 2 HR). Table 1 includes a schedule of enrollment, interventions, and assessments.

**Table 1:** Physical Activity by Technology Help (PATH) schedule of enrollment, interventions, and assessments.

Study’s activities		Study period								
		Enrollment	Allocation	Postallocation					Close-out	
		*-t*_1_	0	*t*_1_	*t*_2_	*t*_3_	*t*_4_	*t*_5_		*TBD*
**Enrollment**										
	Eligibility screen	X								
	Informed consent	X								
	Allocation and wearable installation	X	X							
	Alexa Installation			X						
**Interventions**										
	Control				X	X	X	X		
	Coachtext				X	X	X	X		
	MyCoach				X	X	X	X		
**Assessments**										
	Individual characteristics	X	X							
	Baseline physical activity			X						
	Outcomes				X	X	X	X		
	Analysis and reporting									X

### Participant Inclusion and Exclusion Criteria

Participant inclusion criteria are: (i) adult cancer survivors residing in the State of Maryland who have had one or more of the cancers of interest (breast, prostate, colon, lung, cervical, oral, melanoma); (ii) completed active cancer treatment (surgery, chemotherapy and/or radiation) at least three months prior to enrollment, with the exception of anti-hormonal therapy; (iii) overweight or obese (BMI 25 and above) status; (iv) internet access at home via Wi-Fi; (v) access to an Android or Apple smartphone; (vi) ability to perform low-intensity daily steps (walking), with physician approval; (vii) less than 150 minutes per week of physical activity reported during the previous four weeks; (viii) willingness to wear an accelerometer; and (xi) willingness to consent and accept randomization. Due to disparities of cancer survivorship among minority groups [9,10], the study is focusing the recruitment activities to target African American cancer survivors.

Exclusion criteria are: (i) reported engagement in, or more than, 150 minutes per week of physical activity during the previous four weeks (The Godin-Shephard Leisure-Time Physical Activity Questionnaire) [11-13]; (ii) plans to relocate or travel during the course of the intervention; (iii) stage 4 cancer diagnosis; (iv) current use of a physical activity tracker or engagement in a structured physical activity program; (v) participation in another study that may interfere with our outcome of interest; (vi) an unstable mental condition that would prevent performing the study activities and requirements; and (vii) current or planned pregnancy.

### Innovative Recruitment

Participants are recruited using both passive and active strategies. In the *passive strategy*, the study team distributes flyers at the Johns Hopkins outpatient oncology clinics, patient education rooms, survivorship clinics, and survivorship meetings to spread study awareness among clinic staff, particularly nurse educators and managers. With the *active strategy* for recruitment, the Epic reporting function is used to generate patient lists of those who match the screening criteria at selected Johns Hopkins clinics in Maryland. Screening for existing cancer patients takes place at the outpatient clinics, by specific providers and on follow-up weekly appointments only; potential participants must have a prior diagnosis of a cancer of interest, a BMI of 25 or above, and reside in the state of Maryland. Once a list is generated, the study team actively reaches out to each patient after the outpatient clinic visit to provide them with study information. All candidates identified by either strategy are included in an electronic Clinical Research Management System (CRMS) linked to the EMR. The study coordinator updates the candidate’s status regularly based on candidate eligibility, consent, and enrollment type. Reasons for ineligibility are also recorded in CRMS. Data on the number of eligible patients, along with reasons for not enrolling can be captured and reported to the institution’s Institutional Review Board (IRB). Weekly automated reports can be generated from CRMS to track enrollment and pending statuses.

Once eligibility is determined, an IRB-approved consent designee obtains an IRB-approved written informed consent form (ICF) in the outpatient clinic or at the study office. A signed and dated IRB-approved ICF is documented in a secure participant study file prior to initiating study-related procedures. As part of the consent process, the study team 1) informs each participant of study procedures and requirements and allows sufficient time for the individual to decide whether to participate in the study; 2) answers questions about the details of the study; and 3) ensures that the ICF is approved by the IRB when an amendment to the study protocol is made. Participants are free to withdraw consent for participation in the study at any time, without affecting their current or future treatment.

### Sample Size

The investigators designed this study to compare: (i) a dialogue Intelligent Agent, using data driven Interactive Digital Voice Assist on Amazon Alexa/Echo speaker (*MyCoach*) in assisting participants in increasing their physical activity, with (ii) a text messaging intervention and (iii) a written information/self-motivation intervention. To detect at least a 2000 step difference in means at a SD of 1800 steps/day (the standard deviation of the 28-day daily sample mean is 1800 steps), with a 2-sided alpha of 0.05, and power of 0.8, or 80, 13 participants are required per arm (39 participants total (with Bonferroni correction of 2 comparisons)). To account for intention to treat and possibly per-protocol analysis, the study team subsequently added an additional participant to each arm; therefore, 14 participants are being recruited per arm, for a total of 42 participants. Also, to account for dropout, the protocol also allows the addition of patients to replace those who dropout due to a condition/illness that prevents them from participating in the trial during the run-in period.

### Randomization

Participants are randomized with an allocation ratio of 1:1:1 to the control self-driven arm (Group A), *Coachtext* (Group B), or *MyCoach* (Group C). Once a participant signs the IRB approved ICF, the randomization procedure is conducted. Since the study is recruiting cancer survivors, the team collects data regarding certain prognostic factors that may affect physical performance and thus ability to perform physical activity, including 1) types of cancer; 2) age; and 3) BMI. To attenuate the impact of such factors the study team is using Stratified Permuted Block Randomization, which generates strata by sex, age group, and BMI, then assigns a unique number for each block. At randomization, the PI or study coordinator assign strata by participant variables, then assign the arm after generating a random number out of all possible blocks using random.org. Subsequent assignment is linked to the strata in which the new participant belongs. If the prior block under the selected strata is completed or not started, a new block is selected/opened using random.org. Only the principal investigator (PI) and the study coordinator are designated to conduct the randomization procedure. The PI or the study coordinator report the randomization procedure to the participant orally and record it on the randomization sheet. The study data analyst is blinded to the random assignments.

### Intervention

Those assigned to the control arm (A) receive educational materials and are advised to increase physical activities to 10,000 steps per day. Participants in the control arm receive National Cancer Institute (NCI) printed educational materials providing summary evidence about the benefits of exercise for cancer survivors [14]. While the publication by NCI is meant to encourage participants to become active, it does not provide specific goals or a structured program. As in the other arms, participants in the control arm are expected to use walking as a recommended activity type. Participants in this arm receive the same activity tracker (Fitbit Charge 2 HR) and its companion mobile application as those in the other two study arms.

**Figure 2:**
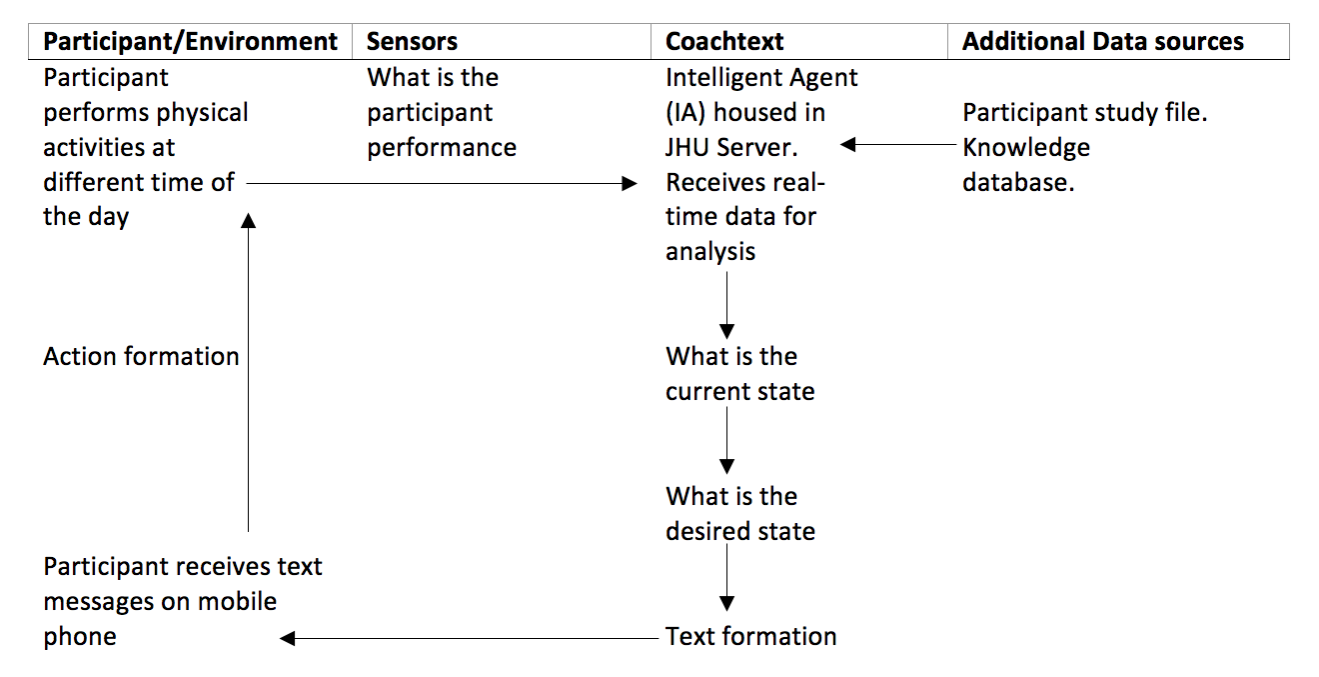
*Coachtext* architecture. JHU: Johns Hopkins University.

Participants in the *Coachtext* arm (B) receive remote coaching using personalized automated data driven text messages, a system designed and developed by the study team that provides text messages to the participant’s cellphone. Those enrolled in this arm provide their cellphone number and complete an online questionnaire about their daily routine, pet ownership, and household information to establish their text messages preferences. The text messages are delivered, based on participant preferences, three times per day for four weeks after the seven-day run-in (baseline data collection). Each message is composed and pushed based on each participant’s real time physical activity performance, taking into account the prerecorded preferences and household information. A sophisticated algorithm reads the participant’s data in the server, then mixes it with the hourly step performance captured by the wearable sensor transmitted to the server, and generates a personalized coaching message. The *Coachtext* algorithm has a flexible goal-based design using data to support its choice on how and when to push a message. The *Coachtext* incoming and outgoing data as well as the algorithm are housed in a server approved by the Institutional Review Board. Figure 2 illustrates the *Coachtext* design.

*Coachtext* is completely automated; however, the study investigators can intervene if needed. This is a precautionary measure that was included in case of system failure or errors, and/or to update the knowledge library of *Coachtext* database. However, no such events have been recorded so far, and no knowledge was changed or newly generated. The study team designed and developed the message content after extensive literature reviews and expert opinions. The messages are formulated based on health behavioral theories with a focus on feedback about actual performance vs expected and habit formation to reach expected performance [15]. Health belief theory is also integrated by offering contents to build knowledge about the benefits of exercise for cancer patients [16]. In addition, the study investigators can check a daily dashboard plotting each participant’s physical activities. The study PI also receive notification in case the system failed to generate or deliver any message on time. As of November 2017, no such event has been recorded. An example of the generated messages includes:

preferred name>, you were too inactive yesterday. You got <#step_count_yesterday steps>. Your goal is 10,000. How about taking a stroll today with <dog_name>. Aim for 10 minutes or more. This will not only be good for you, but is essential to <dog_name's>good health

The third arm is *MyCoach* (C), a conversational Artificial Intelligent Agent delivered via the Amazon Echo home speaker. The investigator chose the Amazon Alexa platform because it has a developer kit that can be utilized to build one’s own voice skill. The study team designed and developed *MyCoach* with knowledge representation, planning, learning, perception, and natural language processing (NLP) using the Alexa developer kit. *MyCoach* uses a sophisticated learning algorithm to enable its functions, and its algorithm uses data from different sources, including, but not limited to the: patient study file, physical activity wearable sensor, Echo home spatial microphones, knowledge library, Amazon music library, intents & responses library, user’s calendar, geo/location data, National Weather Service UV index, and more. *MyCoach* is delivered to the participant through an Amazon Echo speaker [17]. Amazon Alexa was used as an NLP engine to receive the participant’s requests/questions (known as the *Intent*) and the Alexa voice to deliver the answer/advice/coaching (*Response*). In addition, the Alexa companion cellphone application is used to deliver visual responses if needed. The study team designed the *MyCoach* user experience to mimic health coaches. Therefore, the user-initiated request/Intent is the core functionality of *MyCoach*, and no advice/health tip/comments is delivered by *MyCoach* unless the participant requests *MyCoach* advice.

**Figure 3:**
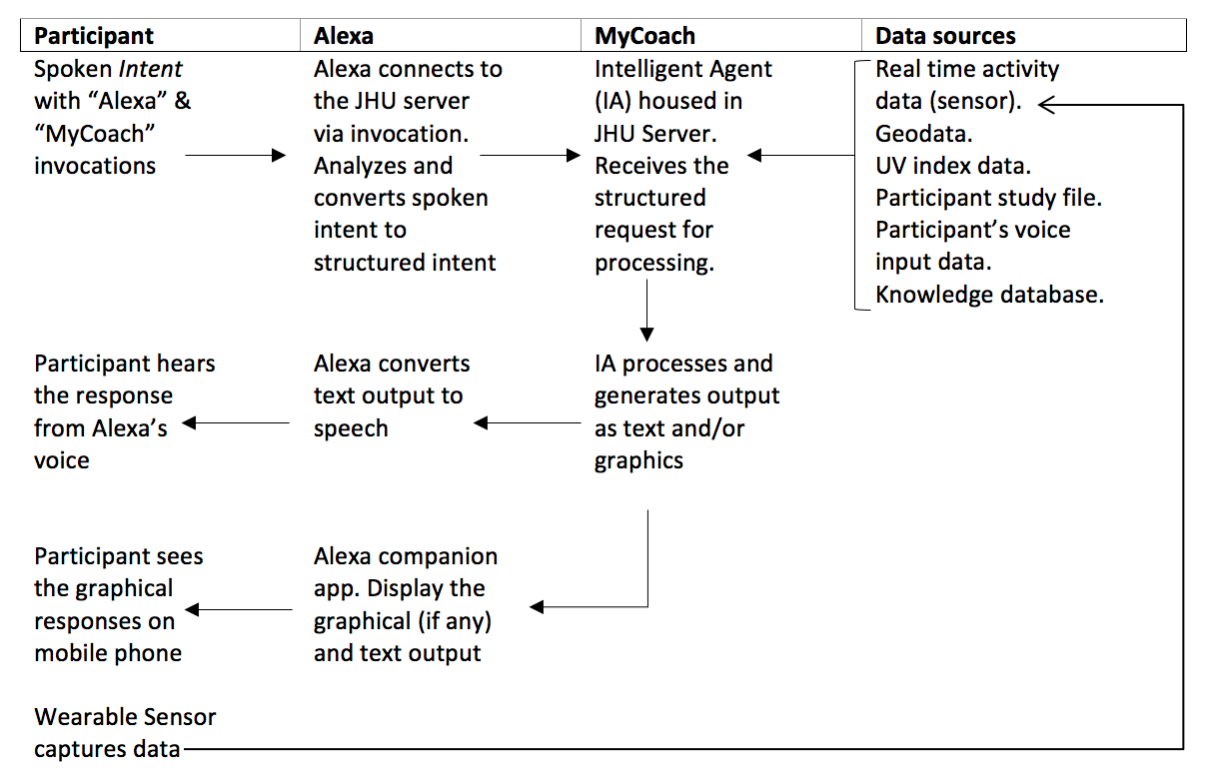
*MyCoach* architecture. JHU: Johns Hopkins University.

The *Intent* has two triggers words (*Invocations*). The first *Invocation* to ignite/turn on Alexa Echo device is “Alexa,” the second, “My coach,” is to alert Alexa that this intent needs to be routed to the *MyCoach* server, within the Johns Hopkins University firewall, to process and generate outputs. For example, the user will say “Alexa, ask My Coach...”, etc. The Alexa NLP engine is used to process the user’s spoken intent into structured representation. The structured request is routed to the *MyCoach* server for processing. During processing, a complex algorithm utilizes multiple data sources and real-time wearable sensor readings to generate a *Response*, which could be a text and/or a graphical response. The servers push the text Response(s) to Alexa, and the graphical responses to the companion app on the user’s cellphone. Alexa converts the text to speech and voices it through the Echo speaker at the user’s home (see Figure 3).

*MyCoach* performs a variety of functions. For example, it offers feedback, assists in formulating habits, provides reminders and alarm, informs the user if he/she need UV skin protection before heading outside, provides health tips and knowledge, and more. Like the participants in *Coachtext*, participants in the *MyCoach* arm also experience a question session to personalize coaching, but in voice conversation format with Alexa/ *MyCoach* **.** All functions are operated by voice, and since hundreds of ways to state the same intent were programmed by the study team to accommodate each different user’s style, no prior training is needed to request each function. An example of intents is as follows:

Alexa, I’m planning to go out for a walk. Ask my coach if I need sunscreen.

User

Alexa, ask my coach, “How is my progress so far?”

User

Alexa, I’m not making good progress; ask my coach for health tips to help me become more active.

User

The study utilized Amazon’s prebuilt user-machine interaction data to track participant utilization of *MyCoach*. Screen shots of the data visualization dashboard used to track interaction by type, time, and intent are provided in Multimedia Appendix 1.

Both interventions (B) and (C) have personalized and tailoring components. Each participant completes a questionnaire about personal family status, habits, pet ownership, work status, and preferred schedule to wake up, eat, work, and relax. All of this information, plus the individual physical performance data, are used to personalize and tailor each individual experience with both interventions. All participants are motivated to increase their physical activities to at least 10,000 steps per day regardless of the arm assignment. Participants in all arms are able to keep the Fitbit Charge 2 HR as a gift from the study team at the end of the trial.

The study team designed each intervention for a specific population, cancer survivors, to best fit their needs, thus the current intervention design is only suitable for such a target population. However, the innovative study design, operations, and technology development make this study easily adaptable for the more general population with slight design modification(s).

### Blinding

Due to the nature of the study interventions, study participants are not blinded. Data for the principal outcomes, however, are collected using sensors. Therefore, the study data analyst is blinded during data collection and during analysis.

### Study Outcomes

The aim of this study is to assess the effectiveness of different technological approaches in increasing physical activity among overweight and/or obese cancer survivors captured by wearable sensors (Fitbit Charge 2 HR). The primary outcome is the percent change in daily steps from the 1-week run-in to the end of the 4-week intervention period. The data for the primary aim is collected in real-time and transferred simultaneously to the computational server of the Johns Hopkins University via an application programming interface (API) that directly transmits the data from the user’s wearable device to the server. Participant wear-time is validated using the heart rate sensor readings on a minute-by-minute basis.

In addition to the primary outcome, the study will examine the following secondary outcomes: 1) total number and duration of activity bouts (defined as 3 minutes or more of uninterrupted activity); 2) fragmentation indices (counting transitions between active/inactive periods) and variability indices (counting the magnitude of transitions between different levels of physical activity); 3) daily diurnal patterns of activity that model patterns in minute-by-minute profiles; and 4) weekly and daily trends of total steps. Levels of physical activity intensity will be defined using each participant’s personalized heart rate reserve.

### Privacy and Safety Aspects

The Johns Hopkins School of Medicine IRB reviewed the protocol, information technology (IT) solutions, safety, and security of the data collection & storage, and approved the study (# IRB00113882). The Sidney Kimmel Comprehensive Cancer Center at Johns Hopkins (SKCCC)/The Clinical Research Review Committee (CRC) also reviewed the protocol and found that this study poses minimal risk to the participants, and assigned Minimum Risk category to this study. SKCCC/CRC recommended forming Data and Safety Monitoring Board for this study & SKCCC/CRC will conduct random audits. To ensure the safety and security of participant information, the *Coachtext* and *MyCoach* were programmed on the Johns Hopkins University server, avoiding the use of commercial servers by Amazon Inc. for public developers. Although the commercial-use free Amazon Web Services server is available with Alexa, it is not compliant with the institutional requirements. However, the study team connected Amazon Echo and Alexa’s voice to the server behind the Johns Hopkins University firewall to protect the user’s privacy. The study also provided a Privacy Policy Statement for the *MyCoach* for the participant who will enable the skills on their Echo devices. This statement explains how data will be shared, stored and processed by Johns Hopkins. The written consent form and the electronic sign-in for each study arm also provide clear descriptions of what data will be collected from each individual and the manner in which it will be used. The study team will assure participants that all automated data collection processes will be stopped after the participant finishes the program or if he/she decides to withdraw from the study. To add another layer of security, *MyCoach* is enabled only for the study participants and not open for public users. Data from the wearable sensors are prescheduled to transfer to our secure server only during the study period. Following the end of the study, the transfer is halted automatically. Regardless of study arm assignment, and in addition to written consent, all users need to complete an online data transfer agreement using their wearable sensor credentials.

### Statistical Analyses

As the primary outcome variable is the 4-week change from the baseline in the average number of steps per day, the primary analysis will use a two-sample t-test to compare each active intervention arm with the control arm based on intent-to-treat strategy. A Bonferroni correction will be used to adjust for multiple comparisons.

For the secondary outcomes (activities bouts, fragmentation, and patterns), a linear regression model will be fitted for the 4-week change from the baseline in the number of steps and duration of activity bouts each day. The independent variables include the baseline number of steps, study arm (modeled by two dummy variables for the two active treatment arms, and the control arm is treated as reference group), and any potential baseline confounding factors. Poisson regression will be used to analyze the total number of activity bouts, fragmentation indices, and variability indices. The independent variables include baseline number of steps, study arm, and any potential baseline confounding factors as the independent variables. In all analyses, data will be properly transformed to fit the model assumption before regression analysis. Although missing data is not anticipated to be a challenge, small amount of non-wear time for charging, bathing, etc. will have minimal effects on the primary study aim since it depends on the average steps across the intervention period. Since Stratified Permuted Block Randomization is being employed, we will consider minimization to balance interventions simultaneously over several prognostic factors to ensure equal distribution across arms. To date nearly 80% of the target population has been enrolled, and only one participant stopped wearing the tracker one day a week for religious reasons.

## Discussion

### Study Rationale

This study designed, developed, and launched a clinical trial to investigate the effect of novel emerging technologies to assist cancer survivors to become more active in their daily lives. The study uses innovative technology and methods to apply autonomous artificial intelligent agents as an intervention. This is an affordable, scalable, and easily deployed personalized and tailored intervention that has the potential to transition from the research setting to general use. It is important to offer a scalable and affordable solution that can assist cancer survivors to become more active. Given the wide general utility of the technology and applicability of the outcome of interest, this approach may also be applicable to the general population with slight modification of the intervention and testing in a larger population study.

The trial operation is minimalist in design and effort. Recruitment activities and enrollment were designed and developed with a focus on low cost and efficiency. The study team developed innovative prescreening in the EMR environment, with remote intervention deployment and data collection processes. The entire trial is operated by the PI and the study coordinator. Data collection processing is autonomous and no interference from the study team or participants is needed. The data flow on a minute-by-minute basis to a structured database for real time analysis allows decision support for the artificial intelligent agent. The same data will be used subsequently to assess the effect of the intervention on the primary outcome. The trial’s recruitment is currently active and eligible consented patients have been enrolled.

Intelligent Agents (IA) are not new. Stuart Russell and Peter Norvig researched this topic extensively in their leading textbook in Artificial Intelligence—Artificial Intelligence: A Modern Approach [18]. Russell and Norvig state that the core behavior of an agent is goal-directed and therefore they refer to agents as “Rational Agents”. These agents can be classified into five classes: simple reflex agents, model-based reflex agents, goal-based agents, utility-based agents, and learning agents [18]. Amazon Alexa and our agent (*MyCoach*) use a combination of different agent classes. The study team chose to use different classes to adapt to the user’s intents.

Artificial intelligence (AI) has promising wide applications in translational research. Enablers to use such technologies, including tools and developer kits, are widely available for interested researchers, while codes libraries are shared among most researchers and powerful platforms are widely used in the form of mobile devices and home speakers. The current study provides insight beyond the target outcomes by its efficient operation. The entire study is operated by 1-2 persons. This is possible because most of the study components, including data collection, have been automated or enhanced by technology solutions. Even the start of the intervention is automated. The study team also automated the instructional communication via scheduled automated emails based on each participant enrollment date. The written consent process is still in paper format, a requirement of the University IRB.

In summary, the current trial demonstrates a very effective and efficient model for intervention that holds the potential to substantially change the way interventions are applied, monitored, and analyzed . The study has been design, developed and launched within the projected cost and timeline. However, challenges still exist. The main challenge for adopting such technologies include understanding the technology’s limitations; how behavioral theories can be adapted to human-machine interaction; limited funding sources for such research; privacy concerns; and the limited aggregable performance metrics for such technologies. Developing such interventions requires in-depth knowledge of AI and their operations, specifically programming. Most of the available behavioral change theories have been developed without consideration of human machine interaction and the potential limitations of such interactions. There are also still concerns about the stability and confidentiality surrounding AI in general, particularly in a health care setting; thus, there is no standardization for adopting AI technologies as there is for traditional interventions. As a result, the design concept for adapting the health coach functions to the AI machine was not straightforward. The user experience aspect was not traditional since no interface currently exists, and voice detection was the main interaction method. It was difficult to orchestrate different components, devices, and algorithms and to develop a secure environment/platform for transferring data within the requirements of the IRB. As a result, it was necessary to build our own server and connect all devices and databases via secure APIs. Further, the study team also overcome the security concerns by building creative solutions including transferring the decision of data transmission and devices communication into the hands of the participants in a form of electronic permissions and unique credentials. Lessons from this study are already helping to develop a more advanced Intelligent Agent to tackle common chronic conditions that rely on self-management and coaching and provide evidence that advancement in technology can be an agent of change to improve health outcomes.

### Trial Status

The study was approved as IRB #IRB00113882 on March 16, 2017. Recruitment activities were initiated in late April 2017. We started with targeted advertisements throughout Johns Hopkins oncology outpatient clinics. We also started active prescreening on Epic EMR to pre-identify participant prior to clinical encounter. The protocol went through a revision to refine the definition of physically active in the inclusion criteria using the American Heart Association definition of physically active. The recruitment activities are expected to last until March 2018. As of January 2018, we screened 75 patients and 39 are already enrolled in the study.

## Appendix

Multimedia Appendix 1 Dashboard for tracking user machine interaction.

Multimedia Appendix 2 Detailed peer review reports and responses by the scientific review committee at the SKCCC.

## Abbreviations

AI: artificial intelligence
API: application programming interface
CRC: Clinical Research Review Committee
CRMS: clinical research management system
EMR: electronic medical records
IA: intelligent agents
ICF: informed consent form
IRB: Institutional Review Board
IT: information technology
JHU: Johns Hopkins University
NCI: National Cancer Institute
NLP: natural language processing
PATH: Physical Activity by Technology Help
PI: principal investigator
SKCCC: Sidney Kimmel Comprehensive Cancer Center
