# Supplementary material for: Increasing Physical Activity Amongst Overweight and Obese Cancer Survivors Using an Alexa-Based Intelligent Agent for Patient Coaching: Protocol for the Physical Activity by Technology Help (PATH) Trial
Source: JMIR Res Protoc. 2018 Feb 12;7(2):e27. doi: 10.2196/resprot.9096 (PMC5826976; doi:10.2196/resprot.9096)

Supplemental Image 1: Dashboard for Tracking User-Machine Interaction

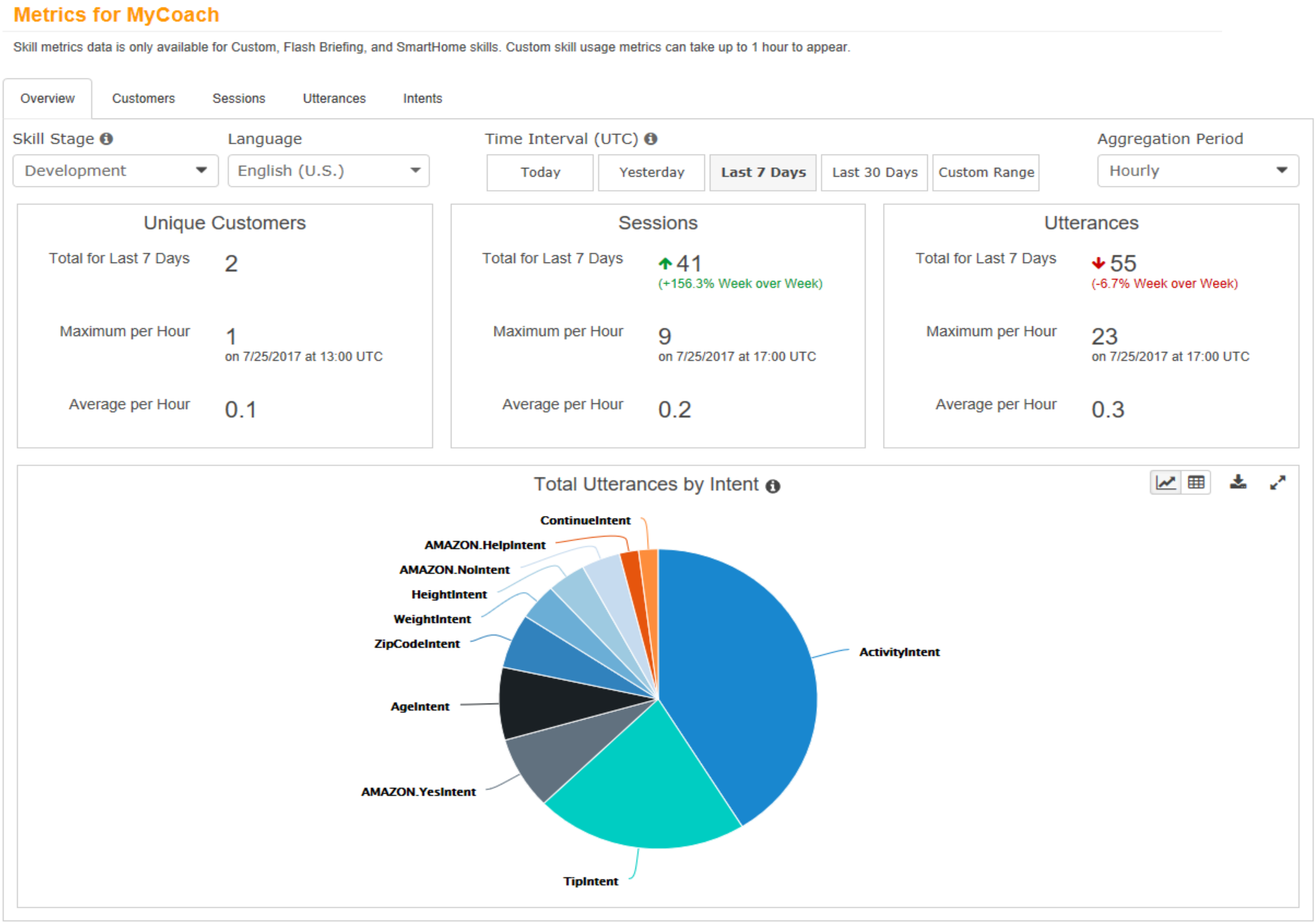

Supplement: Multimedia Appendix 1 [file resprot_v7i2e27_app1.pdf]
