# Supplementary material for: Increasing Physical Activity Amongst Overweight and Obese Cancer Survivors Using an Alexa-Based Intelligent Agent for Patient Coaching: Protocol for the Physical Activity by Technology Help (PATH) Trial
Source: JMIR Res Protoc. 2018 Feb 12;7(2):e27. doi: 10.2196/resprot.9096 (PMC5826976; doi:10.2196/resprot.9096)
Supplement: Multimedia Appendix 2 [file resprot_v7i2e27_app2.pdf]

As an NCI designated Cancer Center, the Sidney Kimmel Comprehensive Cancer Center at Johns Hopkins (SKCCC) is required to review the scientific aspects of all cancer-related clinical research being done at the Institution. The Clinical Research Review Committee (CRC) was established for this purpose.

The CRC is charged with the pre-review of the scientific merit, priorities, and progress of all clinical research involving cancer patients.

In order to ensure compliance with the NCI's guidelines, the following pre-IRB submission process is to be followed for **NEW STUDIES**:

1) All new applications involving cancer-related research will automatically be routed (via eIRB) to the Sidney Kimmel Comprehensive Cancer Center's Clinical Research Office (SKCCC CRO) before submission to the appropriate IRB.

2) The CRO, which provides administrative support for the CRC, will ensure that the submission is complete and schedule its review by the CRC.

    NOTE: If a study does not require review by the CRC, the CRO will forward the study to the IRB.

3) The IRB will not accept a new cancer-related study submission without an exemption from review or an approval by the CRC.

Cancer-related new study applications referred directly to the JHM IRB without CRC review will be forwarded from the IRB to the SKCCC Clinical Research Office (CRO) for CRC review and approval.

4) A copy of the final outcome (approval/disapproval) of each study will be sent to the IRB and the PI for retention.

5) After the study has been reviewed and approved by the CRC, it will then be submitted electronically to the IRB for their review.

In addition to the initial review of a new study, the CRC is required to conduct a continuing review of the scientific progress of all therapeutic cancer trials in conjunction with their IRB mandated continuing renewal. The CRC will notify the IRB of any significant findings.

The CRC meets weekly to review applications. The CRO staff is located in Room 1101 in the 550 Building and may be reached at (410) 955-8866. They can provide details regarding the CRC's review requirements and time frame estimates for completing their review. The CRC may impose stipulations for the conduct of oncology studies at Johns Hopkins; IRB approval would be conditional upon meeting such stipulations.

For more information please visit the CRO website or call (410) 955-8866.

Ahmed Hassoon

From: CRRMC Committee <JHCCCRO@jhmi.edu>
Sent: Thursday, December 29, 2016 10:00 AM
To: Grace Baffoe; Silpa Sharma; Ahmed Hassoon
Cc: Carla Aspril; Jhcccro
Subject: J16162 CRC Sub-Committee Outcome (Requires Revisions)
Attachments: CRC_writeUP.pdf

Follow Up Flag: Follow up
Flag Status: Completed

Dear Dr. Ahmed Hassoon,

J16162 - Novel Individualized Intervention for Behavioral Change Among High-Risk Group Cancer Survivors : physical activities by technology help (PATH).

The Clinical Research Review Subcommittee reviewed J16162 on December 22, 2016

Overall Rating: **Requires Revisions**

Your study cannot be scheduled for CRC review until you respond satisfactorily to the statistical issues listed below.

Note: The CRC is required by the NCI to provide scientific review for all cancer-related research. The protocol cannot be scheduled for CRC review until we receive a response to all Subcommittee comments. Please send a cover letter with responses to all PURPLE comments and a tracked changes version of all revised documents.

If you have any questions about the statistical component of the review, please contact Oncology Biostatistics at 5-4884.

Your comments can be viewed below and online.
https://apps.onc.jhmi.edu/researchapps/crc/actions/loadProtocol.cfm?protocolID=6340

Thanks.

Please contact us if you have any questions:

JHCCCRO@jhmi.edu
Phone: 410-955-8866
Fax: 410-614-1328

Comments

Forum Comment Color Legend Sub-Committee Reply Requested Question Answered

Sub-Committee

The ICTR is available to assist investigators with every aspect of planning/running any study. Please contact the ICTR Research Navigators, who are prepared to assist with any question related to clinical or even pre-clinical translational research by submitting an online “ICTR Connection Request” through the ICTR’s “Ask the Research Navigator” at http://ictr.johnshopkins.edu/consulting/consulting-services/ask-the-research-navigator-2.

Carla Aspril 12/29/16 @ 9:58 AM

The description of the study procedure is inconsistent. In some place (2nd paragraph on page 2), it says that the procedure includes a one-week run-in followed by a four-weeks intervention. At the bottom of the same page, it says that the procedure includes a two-week run-in followed by a two-week intervention. In the recruitment, it says "Research staff will obtain consent in the pre-operative clinic or in the holding area on the day of surgery or on the floor prior to surgery". How could these patients complete the desired 5-week exercise if they are going to receive surgery after being enrolled into the study?

Peng Huang 12/22/16 @ 5:56 PM

What randomization method will be used? Should it be performed before or after the run-in period? If performed before the run-in, then the adjustment to the baseline difference should be included in the analysis. Could Amazon Echo Alexa count the number of steps? Would it mean that steps performed outside the Wi-Fi range will not be counted? The data analysis needs to adjust for imbalance of the baseline steps. Please consult the study statistician to improve the presentation of the protocol.

Peng Huang 12/22/16 @ 5:56 PM

How the primary endpoint will be measured since the total number of steps varies from day to day?

Peng Huang 12/22/16 @ 5:56 PM

No imaging performed on study.

Jeffrey Leal 12/22/16 @ 8:04 AM

QA – Protocol (audit/monitoring): All interventional cancer center trial classified as institutional or externally-peer-reviewed and authored by JHU SKCCC must adhere to all JHU SKCCC SOPs – http://cro.onc.jhmi.edu/. PI is responsible for internally monitoring the study and establishing additional external data & safety monitoring oversight, as required. All safety reporting will be assessed at least annually by the SKCCC Safety Monitoring Committee (SMC); requested source documentation, including SKCCC-required AE & Deviation Logs, must be continually updated per GCP and provided annually for peer-review. PI is responsible for internally monitoring study and establishing additional external data & safety monitoring oversight (MEC, DSMB, etc). SMC requires that all DSMP Level I-IV study teams maintain a cumulative, up-to-date study-specific master adverse event log and a protocol deviation log (in Excel format) for peer-review of study’s safety profile. Templates available here: http://cro.onc.jhmi.edu/. If the JHU study team is the Coordinating Center (CC) then participating sites must also be required to maintain versions of these logs, and submit them the CC team upon request. Contact the CRO QA Office (croqaoffice@jhmi.edu) with questions.

Courtney Wheeler 12/20/16 @ 4:49 PM

LEVEL 1: This is a DSMP Level I per the JHU SKCCC DSMP. The following language should appear in the protocol: “The PI is responsible for internally monitoring the study and establishing additional external data & safety monitoring oversight, as required. The PI will also monitor the progress of the trial, review safety reports, and confirm that the safety outcomes and response assessments favor continuation of the study.” CROQA will perform risk-based auditing, depending on rate of accrual and relevant review findings. Review reporting will assessed annually by the SKCCC Safety Monitoring Committee (SMC); requested source documentation, including SKCCC-required AE & Deviation Logs, must be continually updated per GCP and provided annually to assure validity of data and safety of subjects for peer-review. The PI is also responsible for establishing additional external data & safety monitoring oversight (MEC, DSMB, etc) as required per protocol.” Contact the CRO QA Office (croqaoffice@jhmi.edu) with questions.

Courtney Wheeler 12/20/16 @ 4:27 PM

As an NCI-designated Comprehensive Cancer Center, we are required to post certain study materials on an NCI website as part of the Clinical Trials Reporting Program (“CTRP”). Unfortunately, sometimes our industry partners object to the posting of our investigator-initiated protocols on the grounds that they include proprietary information about their products. Despite this, the NCI does not consider these to be “industry” studies, so we generally cannot avoid publication of the protocol materials. Can you please review your protocol and ICF documents for the study referenced above, and let us know whether or not these include any proprietary (nonpublic) information you received from the industry sponsor? Typically, if there is any such info, it would likely relate to industry-supplied materials (drugs/devices) and be included in the protocol.

caitlin joffe 12/19/16 @ 2:21 PM

FYI: The CRO will register this trial with CTRP (Clinical Trials Registration Program), which requires submitting the full protocol for NCI use only. Please acknowledge.

caitlin joffe 12/19/16 @ 2:21 PM

Upload the newest version of the HIPAA Form 4 vs 7/14 (section 11). http://cro.onc.jhmi.edu/?page=280

Carla Aspril 12/16/16 @ 10:44 AM

When you are ready for the study to be posted on the CRO Protocol Library (after IRB approval and PRA approval, if required), please download the "Protocol Library Posting Checklist" from the CRO website, complete, and email back to the CRO along with requested docs/data. After the study is posted, the CRO will change the study status to "Active" in CRMS.

Carla Aspril 12/16/16 @ 10:42 AM

The CRO and IRB have developed standard eIRB language to avoid having applications returned as incomplete. Please view this language on our website and incorporate into your application if needed. http://cro.onc.jhmi.edu/?page=283

Carla Aspril 12/16/16 @ 10:41 AM

The study staff is required to enter enrollment data in CRMS for all studies enrolling Cancer Center subjects. Please confirm. (If you do not have access to CRMS, please go to https://lms.learnshare.com/authenticate/shib/default.asp to complete the CRMS training course. For additional help or training, contact CRMSHelp@jhmi.edu

Carla Aspril 12/16/16 @ 10:41 AM

Clinical Research Office
Baltimore, MD 21205
phone: 410-955-8866
fax: 410-614-1328
email: JHCCCRO@jhmi.edu

WARNING: E-mail sent over the Internet is not secure. Information sent by e-mail may not remain confidential.

DISCLAIMER: This e-mail is intended only for the individual to whom it is addressed. It may be used only in accordance with applicable laws. If you received this e-mail by mistake, notify the sender and destroy the e-mail.

From: Ahmed Hassoon, MD, MPH, PMP

To: The Clinical Research Review Subcommittee

Subject: Responses to the review of J16162 on December 22, 2016

1- Carla Aspril 12/29/16 @ 9:58 AM: Thank you, we will utilize very available resources to ensure the success of this study.
2- Peng Huang 12/22/16 @ 5:56 PM: Sorry for the mistake. The study is 1 week run-in and 4 weeks of intervention. We will recruit and consent at the **outpatient clinic**. Only cancer survivors who completed the active treatment (including surgery) at least three months before will be eligible to participate.
3- Peng Huang 12/22/16 @ 5:56 PM: Randomization take place before run-in. Run-in is a one week monitor to establish baseline for each participant in the three arms. The steps will be capture by accelerometer and come to our server on minute by minute cases from the accelerometer manufacture. We obtained API and already received approval for that access. Alexa get the steps from our server and not from the user. Steps recording in our server start immediate after randomization once the accelerometer is activated at the same occasion. There is no transfer of steps between the user and Alexa. The accelerometer sends the data to the user's phone, the user's phone sends it to Fitbit, Fitbit send it to us in a fast stream process. Alexa will access our server with the steps when the user invocates Alexa. We are planning to measure variation of baseline across arm, and we will adjust for imbalance. Please see the diagram below:

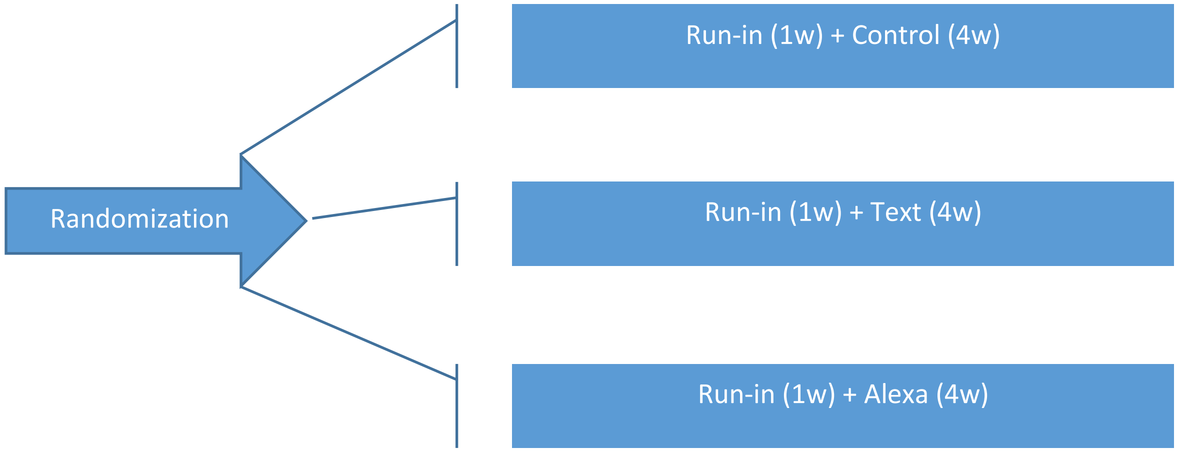

The intervention for text and Alexa will be scheduled to start on the 8th days after day 1 of run-in. However, the data capture will start on day 1. The intervention will be triggered remotely for text, and by user invocation of Alexa on the 8th day.

Participants will be randomized to one for the three following arms:
- Self-Directed - Smart texting - Alexa (My coach)
We will randomize participants to 1:1:1 stratified by gender. To prevent prediction of randomization, we will generate the randomization schedule in variable blocks sizes. Randomization will take place at the outpatient clinic after the study team confirms that all screening activities have occurred, individual meets all eligibility criteria, and all needed data have been collected. Individuals who do not meet eligibility will not be randomized. All staff members who will conduct randomization are not aware of the data collection since we design the data collection system to be automated and remotely without patients or study staff involvement/awareness.

4- Peng Huang 12/22/16 @ 5:56 PM: Thank you. Through our interventions, we are aiming to help cancer survivors to consistently perform on average 10,000 steps/day. We will measure average across all days, weekly and daily trends. We will utilize the oncology biostatistics resources to improve that section. We also have professor in applied mathematics in our team who will help us during the analysis.
5- Courtney Wheeler 12/20/16 @ 4:49 PM: Thank you. We will adhere to all the institutional requirements.
6- Courtney Wheeler 12/20/16 @ 4:27 PM: We will add it to the protocol.
7- caitlin joffe 12/19/16 @ 2:21 PM: **We did not receive any industrial support or any non-public materials**. All the work is being done by our own staff and our own resources. We are using public API(s) and public developer kit to program Alexa remotely.
8- caitlin joffe 12/19/16 @ 2:21 PM: Yes. However, we suggest to wait for the final revised protocol since we received several comments to improve the presentation in the protocol.
9- Carla Aspril 12/16/16 @ 10:44 AM: We will do so. **However, the link is broken.**
10- Carla Aspril 12/16/16 @ 10:42 AM: We will do so.
11- Carla Aspril 12/16/16 @ 10:41 AM: We will utilize every available recourse. However, **the link is broken.**
12- Carla Aspril 12/16/16 @ 10:41 AM: I do have access to CRMS under a previous trial. Do I need a new access? **Certificate attached**

Ahmed Hassoon

From: CRRMC Committee <JHCCCRO@jhmi.edu>
Sent: Friday, January 13, 2017 12:57 PM
To: Grace Baffoe; Silpa Sharma; Ahmed Hassoon
Cc: Carla Aspril; Jhcccro
Subject: J16162 CRC Sub-Committee Outcome (Requires Revisions)
Attachments: CRC_writeUP.pdf

Dear Dr. Ahmed Hassoon,

J16162 - Novel Individualized Intervention for Behavioral Change Among High-Risk Group Cancer Survivors physical activities by technology help (PATH).

The Clinical Research Review Subcommittee reviewed J16162 on January 12, 2017

Overall Rating: **Requires Revisions**

Your study cannot be scheduled for CRC review until you respond satisfactorily to the statistical issues listed below.

Note: The CRC is required by the NCI to provide scientific review for all cancer-related research. The protocol cannot be scheduled for CRC review until we receive a response to all Subcommittee comments. Please send a cover letter with responses to all PURPLE comments and a tracked changes version of all revised documents.

If you have any questions about the statistical component of the review, please contact Oncology Biostatistics at 5-4884.

Your comments can be viewed below and online.
https://apps.onc.jhmi.edu/researchapps/crc/actions/loadProtocol.cfm?protocolID=6354

Thanks.

Please contact us if you have any questions:

JHCCCRO@jhmi.edu
Phone: 410-955-8866
Fax: 410-614-1328

Comments

Forum Comment Color Legend Sub-Committee Reply Requested Question Answered

Sub-Committee

In the revised protocol, there is no data analysis plan for both primary and secondary endpoints. Also, no information is giving about what text message or voice script will be used to encourage participants to increase the physical activities. Since the PI stated that the study will utilize the oncology biostatistics resources to improve that section, please submit a service request to oncology biostatistics to get help from one of the biostatisticians

Peng Huang 1/13/17 @ 11:33 AM

Response is satisfactory.

Carla Aspril 1/12/17 @ 8:41 AM

QA: No comment/concerns.

Courtney Wheeler 1/5/17 @ 11:52 AM

If you consider the protocol document/ICF to contain proprietary information you must provide a redacted copy of the initial protocol/ICF submission and each amendment thereafter. Redacted documents should be emailed to the CRO.

caitlin joffe 1/3/17 @ 9:15 AM

A reply has not been provided to the initial review. Please reply/confirm: As an NCI-designated Comprehensive Cancer Center, we are required to post certain study materials on an NCI website as part of the Clinical Trials Reporting Program ("CTRP"). Unfortunately, sometimes our industry partners object to the posting of our investigator-initiated protocols on the grounds that they include proprietary information about their products. Despite this, the NCI does not consider these to be "industry" studies, so we generally cannot avoid publication of the protocol materials. Can you please review your protocol and ICF documents for the study referenced above, and let us know whether or not these include any proprietary (nonpublic) information you received from the industry sponsor? Typically, if there is any such info, it would likely relate to industry-supplied materials (drugs/devices) and be included in the protocol.

caitlin joffe 1/3/17 @ 9:15 AM

A reply has not been provided to the initial review. Please reply/confirm: FYI: The CRO will register this trial with CTRP (Clinical Trials Registration Program), which requires submitting the full protocol for NCI use only. Please acknowledge.

caitlin joffe 1/3/17 @ 9:15 AM

Clinical Research Office
Baltimore, MD 21205
phone: 410-955-8866
fax: 410-614-1328
email: JHCCCRO@jhmi.edu

WARNING: E-mail sent over the Internet is not secure. Information sent by e-mail may not remain confidential.

DISCLAIMER: This e-mail is intended only for the individual to whom it is addressed. It may be used only in accordance with applicable laws. If you received this e-mail by mistake, notify the sender and destroy the e-mail.

From: Ahmed Hassoon, MD, MPH, PMP

To: The Clinical Research Review Subcommittee

Subject: Responses to the review of J16162 on January 17, 2017

1- Peng Huang 1/13/17 @ 11:33 AM: Thank you, we revised the statistical section and submitted oncology biostatistics request to assist in refining the statistical plan. Professor Dan Naiman provided review for that section and he will provide an ongoing support to our statistical plan during analysis. I also attached sample bank of messages that we will use in the intervention (voice and text).
2- caitlin joffe 1/3/17 @ 9:15 AM: No proprietary information in the protocol.
3- caitlin joffe 1/3/17 @ 9:15 AM: No proprietary information in the protocol or the ICF documents. We don't have industrial partner. We are using a consumer products and programming our own application using these platforms. We don't have any support from any of the mentioned industry even technical support. Nothing in our protocol or ICF document considered nonpublic information. All devices that we are using are readily available for public as consumer devices.
4- caitlin joffe 1/3/17 @ 9:15 AM: As we indicated before, YES we acknowledge.

Ahmed Hassoon

From: CRRMC Committee <JHCCCRO@jhmi.edu>
Sent: Friday, January 27, 2017 12:40 PM
To: Grace Baffoe; Silpa Sharma; Ahmed Hassoon
Cc: Carla Aspril; Jhcccro
Subject: J16162 CRC Sub-Committee Outcome (Requires Revisions)
Attachments: CRC_writeUP.pdf

Dear Dr. Ahmed Hassoon,

J16162 - Novel Individualized Intervention for Behavioral Change Among High-Risk Group Cancer Survivors physical activities by technology help (PATH).

The Clinical Research Review Subcommittee reviewed J16162 on January 26, 2017

Overall Rating: **Requires Revisions**

Your study cannot be scheduled for CRC review until you respond satisfactorily to the statistical issues listed below.

Note: The CRC is required by the NCI to provide scientific review for all cancer-related research. The protocol cannot be scheduled for CRC review until we receive a response to all Subcommittee comments. Please send a cover letter with responses to all PURPLE comments and a tracked changes version of all revised documents.

If you have any questions about the statistical component of the review, please contact Oncology Biostatistics at 5-4884.

Your comments can be viewed below and online.
https://apps.onc.jhmi.edu/researchapps/crc/actions/loadProtocol.cfm?protocolID=6393

Thanks.

Please contact us if you have any questions:

JHCCCRO@jhmi.edu
Phone: 410-955-8866
Fax: 410-614-1328

Comments

Forum Comment Color Legend

Sub-Committee

The study statistics section is still not complete. The proposed t-test and analysis of variance cannot adjust for baseline confounders. Please work with the study statistician to modify this section.
Peng Huang 1/27/17 @ 10:49 AM

Clinical Research Office
Baltimore, MD 21205
phone: 410-955-8866
fax: 410-614-1328
email: JHCCCRO@jhmi.edu

WARNING: E-mail sent over the Internet is not secure. Information sent by e-mail may not remain confidential.

DISCLAIMER: This e-mail is intended only for the individual to whom it is addressed. It may be used only in accordance with applicable laws. If you received this e-mail by mistake, notify the sender and destroy the e-mail.

Ahmed Hassoon

From: CRC Committee <JHCCCRO@jhmi.edu>
Sent: Thursday, February 9, 2017 2:23 PM
To: Grace Baffoe; Silpa Sharma; Ahmed Hassoon
Cc: Jhcccro; Carla Aspril
Subject: J16162 CRC (Return to Committee Chair or Designee for Re-Review) / Subcommittee (Satisfactory)
Attachments: J16162.pdf

Follow Up Flag: Follow up
Flag Status: Flagged

Dear Dr. Ahmed Hassoon,

J16162 Novel Individualized Intervention for Behavioral Change Among High-Risk Group Cancer Survivors physical activities by technology help (PATH)

Attached please find the CRC's outcome letter for J16162, which was reviewed on 2/9/2017.

- **CRC Outcome**:Return to Committee Chair or Designee for Re-Review
- **Subcommittee Outcome**: Satisfactory
- **Comments**: below and on the forum - https://apps.onc.jhmi.edu/researchApps/crc/actions/loadProtocol.cfm?protocolID=6403

NOTE: The application has been returned in EIRB; however, you must reply via email with a response letter and a tracked changes copy of all revised documents. Please wait until your response receives approval to resubmit the corrected application.

If you have any questions please feel free to contact the CRO at 5-8866 or via email.

Tips for replying:

- PURPLE and ORANGE comments require response.
- GREEN and WHITE comment do NOT require a response.
- Click Reply and type your response directly below each comment. You can save your draft or forward to your team members for their input.
- To have your comments automatically show in a different color, go into email Tools › Options › Preferences Tab › Email Options › Check Mark my comments with. Then type in text to appear next to your comment.

Forum Comment Color Legend ☐ Sub-Committee ☐ Reply Requested ☐ Question Answered

Scientific Merit

Will patients who don't provide an email address be eligible to participate in the study? Will they be assigned to arms that don't include the Echo? What are you having the Echo say to those enrolled? Can you give us examples of the messages? How do you control this?
Richard Ambinder 2/8/17 @ 10:02 PM

Study Design

This is an interesting pilot study to evaluate the most adequate way to implement a physical activity goal in cancer survivors. It randomizes to either a self directed approach, smart texting or Alexa My Coach and the objective after one

week of run in is to reach documented 10,00 steps/day. Patients are startified for age group,gender ,BMI. The flow of the study is post 4 weeks is not very clearly outlined. Also it is unclear how the outcome (s) will be evaluated. Is this part of a long term plan for survivors? It may be important to establish a reasonable disease (types of cancer, extent of disease etc) and prior treatment (chemo therapy,radiation hormones etc) criteria in the stratification to minimize randomization imbalance,
Mario Eisenberger 2/7/17 @ 3:58 PM

Sub-Committee

QA – Protocol (audit/monitoring): All interventional cancer center trial classified as institutional or externally-peer-reviewed and authored by JHU SKCCC must adhere to all JHU SKCCC SOPs – http://cro.onc.jhmi.edu/. PI is responsible for internally monitoring the study and establishing additional external data & safety monitoring oversight, as required. All safety reporting will be assessed at least annually by the SKCCC Safety Monitoring Committee (SMC); requested source documentation, including SKCCC-required AE & Deviation Logs, must be continually updated per GCP and provided annually for peer-review. PI is responsible for internally monitoring study and establishing additional external data & safety monitoring oversight (MEC, DSMB, etc). SMC requires that all DSMP Level I-IV study teams maintain a cumulative, up-to-date study-specific master adverse event log and a protocol deviation log (in Excel format) for peer-review of study's safety profile. Templates available here: http://cro.onc.jhmi.edu/. If the JHU study team is the Coordinating Center (CC) then participating sites must also be required to maintain versions of these logs, and submit them the CC team upon request. Contact the CRO QA Office (croqaoffice@jhmi.edu) with questions.
Courtney Wheeler 2/1/17 @ 3:34 PM

QA: LEVEL 1: This is a DSMP Level I per the JHU SKCCC DSMP. The following language should appear in the protocol: "The PI is responsible for internally monitoring the study and establishing additional external data & safety monitoring oversight, as required. The PI will also monitor the progress of the trial, review safety reports, and confirm that the safety outcomes and response assessments favor continuation of the study." CROQA will perform risk-based auditing, depending on rate of accrual and relevant review findings. Review reporting will assessed annually by the SKCCC Safety Monitoring Committee (SMC); requested source documentation, including SKCCC-required AE & Deviation Logs, must be continually updated per GCP and provided annually to assure validity of data and safety of subjects for peer-review. The PI is also responsible for establishing additional external data & safety monitoring oversight (MEC, DSMB, etc) as required per protocol." Contact the CRO QA Office (croqaoffice@jhmi.edu) with questions.
Courtney Wheeler 2/1/17 @ 3:33 PM

Per Peng Huang: The statistical issues have been resolved, and I gave a pass to the protocol.
Carla Aspril 2/1/17 @ 11:12 AM

CRO

Clinical Research Office
Sidney Kimmel Comprehensive Cancer Center
Baltimore, MD 21205
phone: 410-955-8866
fax: 410-614-1328
email: JHCCCRO@jhmi.edu

JOHNS HOPKINS
MEDICINE
THE SIDNEY KIMMEL COMPREHENSIVE CANCER CENTER

Clinical Research Review Committee
Sidney Kimmel Comprehensive Cancer Center
at Johns Hopkins University
550 North Broadway
Room 1003
Baltimore, Maryland 21205
(410) 955-8866

DATE: Thursday, February 9, 2017

TITLE: J16162 Novel Individualized Intervention for Behavioral Change Among High-Risk Group Cancer Survivors physical activities by technology help (PATH)

PRINCIPAL INVESTIGATOR: Dr. Ahmed Hassoon

OUTCOME: Return to Committee Chair or Designee for Re-Review

The Clinical Research Review Committee (CRC) reviewed the above noted study on Thursday, February 9, 2017 The Committee had some concerns that are documented on the CRC Forum.

To view comments on the CRC Forum made by the reviewers please access the CRC Forum. Items highlighted in orange or purple will require a response.

Upon receipt of your response, the CRC Designee will re-review your protocol.

Mario Eisenberger, MD, Chair
SKCCC Clinical Research Review Committee

CRC new comments and responses:

Scientific Merit

Will patients who don't provide an email address be eligible to participate in the study? Will they be assigned to arms that don't include the Echo? What are you having the Echo say to those enrolled? Can you give us examples of the messages? How do you control this?
Richard Ambinder 2/8/17 @ 10:02 PM

Response:

Thank you for your comments. Please see our responses below:
Will patients who don't provide an email address be eligible to participate in the study? Will they be assigned to arms that don't include the Echo? Any patient who will not provide an email address will not be able to participate. The email requirement goes beyond Echo device registration. All participant must have email to install and register the accelerometer (Fitbit and its app) under their name. The email act as authentication signature that link the device, the app, the data to the participant. This is how we obtain permission from Fitbit to access the participant data on real-time. So email is needed for control, text and My Coach.

What are you having the Echo say to those enrolled? Can you give us examples of the messages? How do you control this?

Echo is the speaker name; Alexa is the Digital voice assist. My Coach is our own health digital voice assist. My Coach use Alexa as a medium to communicate with the patient. My coach will have certain functions based on patient's request (intent). And we have programed more than 2000 ways of asking/intent to make My Coach understand different speaking/grammar style. My Coach will not do anything unless the patient request and activate the functions that we will offer. We design thousands of different ways to ask the same question, and hundreds of answers based on pre-defined inputs. Inputs are number of steps, what time of the day the patient is having the conversation with My Coach, patient unique variables, workout preference, exercise music style. My Coach use logic driven artificial intelligence and not unsupervised intelligence. It will only say what we pre-program it to say. Please ignore punctuation. My Coach read sentence in a different way that what we do.

The functions are:

1- Checking and reminder of steps performance. These are short sentences about the patient's performance, advices and encouragement to be active and hit the daily goal of 10k steps. For example:
   If the patient asked My Coach on day three during the morning hours. My Coach will check yesterday steps, if they are <7500. My coach will say
   "*you were too sedentary yesterday. Everyone needs a day off now or then or has a personal issue come up. Try to be much more active today to reach your third day goal of 30,000 steps. Turn talking on the phone into an activity. For every 20 minutes you're on the phone, you could accumulate another 2,000 steps or more! Even pacing back and forth down a hallway counts!*"

Another function is working-out at home in a form of dancing. For example, playing music for working out at home. My Coach has access to millions of song and music that can be played at request. Especially during cold weather. Echo speaker has surround sound and very powerful sound to have a party at home despite the small size.

Another function is weather. My Coach can check the weather and give forecast to the patient before going out. We are also working to access EPA server to give UV index by zip code so the patient can get advice about sun screen before heading out for a walk. As an example if the UV is 7-9 My Coach will say:

"*The U V light is high*
Protection against sun damage is needed. Wear a wide-brimmed hat and sunglasses, use sunscreen SPF 30+ and wear a long-sleeved shirt and pants when practical."

Another function is the healthy tips and steps incorporation in daily life. All invoked by patient intent. These messages have some element of personalization. If a person has a daily job or a car the messages adapt to that. We have a message bank , My Coach will use the bank to voice these messages

"Exercise during all stages of life is important, but it can be particularly important for cancer survivors because it can help you feel better after treatment and promote survivorship. Studies show that walking 30 minutes every day can lower the chances of cancer recurrence and death by up to 40 percent"

'While at work, walk down the hall to speak with someone rather than using the telephone."

"Take the stairs instead of the elevator. Or get off a few floors early and take the stairs the rest of the way"

"How many times have you circled the parking lot to find the closest spot? Spare yourself the stress and gain more energy by parking far away or even in a remote lot and walking farther to your destination. < for car owner only>"

"Participating in cancer awareness walks is a great way to exercise and help other cancer survivors. Search for local events and invite family and friends to join you."

Study Design

This is an interesting pilot study to evaluate the most adequate way to implement a physical activity goal in cancer survivors. It randomizes to either a self directed approach, smart texting or Alexa My Coach and the objective after one week of run in is to reach documented 10,00 steps/day. Patients are startified for age group,gender ,BMI. The flow of the study is post 4 weeks is not very clearly outlined. Also it is unclear how the outcome (s) will be evaluated. Is this part of a long term plan for survivors? It may be important to establish a reasonable disease (types of cancer, extent of disease etc) and prior treatment (chemo therapy,radiation hormones etc) criteria in the stratification to minimize randomization imbalance,

Mario Eisenberger 2/7/17 @ 3:58 PM

Response:

Thank you very much. Excellent points. Here are our responses

It randomizes to either a self-directed approach, smart texting or Alexa My Coach and the objective after one week of run in is to reach documented 10,00 steps/day

The target is actually 10,000 steps/day, which is the minimum recommended steps per day by the American Heart Associate. This translate to 30 minutes of activity per day.

The flow of the study is post 4 weeks is not very clearly outlined.

The study is a pilot randomized controlled trial with three arms. Our primary aim is to examine if such novel technology can help cancer survivors to increase their daily physical activities and reach 10k per day (average). All arms will have one week of run it to establish baseline average number of steps (no intervention). We do have physical activity screener, but we want to capture the actual number of steps to make sure we get an accurate measure.

Following the run in (one week). The intervention will start. We have self-drive, text, or My Coach. He technology will encourage the patient to do 10k/day for the next 4 weeks of intervention. The intervention effect is the average number of steps during the 4 weeks. We will do within and between arms analyses. Our study is powered to detect a difference of 2000 steps with SD of 1800 steps. We also have other secondary outcomes that will analyses the granularity of activities during the 4 weeks, including bouts of activity and inactivities, trends, acceleration, time spending active, sitting and more. All these data come to our server in real time for analysis. We will also examine if the frequency of interaction with technology associated with increase in physical activities.

Is this part of a long term plan for survivors?

Yes. We are piloting the technology on physical activities. But we are thinking of adding more functions in the near future. For example, weight control, graving control, health diet tips, medication adherence, depression screener, Q&A about cancer specific survivorship challenges and solutions …etc

It may be important to establish a reasonable disease (types of cancer, extent of disease etc) and prior treatment (chemo therapy,radiation hormones etc) criteria in the stratification to minimize randomization imbalance,

Excellent point. The CRF grant focus on six cancers in the state of Maryland. Prostate, Breast, Colon, Lung, Cervical, Skin, and Oral. We understand that each cancer and its related management have effect on physical performance. We will do our best to have equal distribution across the arms. Prostate cancer and testosterone is a good example of how it may affect patient performance based on muscle mass. We will control for this effect during randomization and our statistical approach. For example, in the randomization we will use Stratified Permuted Block Randomization, and we will consider Minimization to balance interventions simultaneously over several prognostic factors.

Sub-Committee

QA – Protocol (audit/monitoring): All interventional cancer center trial classified as institutional or externally-peer-reviewed and authored by JHU SKCCC must adhere to all JHU SKCCC SOPs – http://cro.onc.jhmi.edu/. PI is responsible for internally monitoring the study and establishing additional external data & safety monitoring oversight, as required. All safety reporting will be assessed at least annually by the SKCCC Safety Monitoring Committee (SMC); requested source documentation, including SKCCC-required AE & Deviation Logs, must be continually updated per GCP and provided annually for peer-review. PI is responsible for internally monitoring study and establishing additional external data & safety monitoring oversight (MEC, DSMB, etc). SMC requires that all DSMP Level I-IV study teams maintain a cumulative, up-to-date study-specific master adverse event log and a protocol deviation log (in Excel format) for peer-review of study's safety profile. Templates available here: http://cro.onc.jhmi.edu/. If the JHU study team is the Coordinating Center (CC) then participating sites must also be required to maintain versions of these logs, and submit them the CC team upon request. Contact the CRO QA Office (croqaoffice@jhmi.edu) with questions.

Courtney Wheeler 2/1/17 @ 3:34 PM

QA: LEVEL 1: This is a DSMP Level I per the JHU SKCCC DSMP. The following language should appear in the protocol: "The PI is responsible for internally monitoring the study and establishing additional external data & safety monitoring oversight, as required. The PI will also monitor the progress of the trial, review safety reports, and confirm that the safety outcomes and response assessments favor continuation of the study." CROQA will perform risk-based auditing, depending on rate of accrual and relevant review findings. Review reporting will assessed annually by the SKCCC Safety Monitoring Committee (SMC); requested source documentation, including SKCCC-required AE & Deviation Logs, must be continually updated per GCP and provided annually to assure validity of data and safety of subjects for peer-review. The PI is also responsible for establishing additional external data & safety monitoring oversight (MEC, DSMB, etc) as required per protocol." Contact the CRO QA Office (croqaoffice@jhmi.edu) with questions.

Courtney Wheeler 2/1/17 @ 3:33 PM

Answer: Thank you. During our previous review, we indicated that we will comply with all JHU SKCCC requirement, including the adherence to SKCCC SOP.

Answer: Thank you. During our previous review we indicated out agreement with this requirement. Please see page 7 section 8.

Per Peng Huang: The statistical issues have been resolved, and I gave a pass to the protocol.
Carla Aspril 2/1/17 @ 11:12 AM

JOHNS HOPKINS
MEDICINE
THE SIDNEY KIMMEL
COMPREHENSIVE CANCER
CENTER

Clinical Research Review Committee
Sidney Kimmel Comprehensive Cancer Center
at Johns Hopkins University
550 North Broadway
Room 1003
Baltimore, Maryland 21205
(410) 955-8866

DATE: Monday, February 20, 2017

TITLE: J16162 Novel Individualized Intervention for Behavioral Change Among High-Risk Group Cancer Survivors physical activities by technology help (PATH)

PRINCIPAL INVESTIGATOR: Dr. Ahmed Hassoon

OUTCOME: Forward to IRB

The Clinical Research Review Committee (CRC) designee reviewed and approved the above noted protocol on Monday, February 20, 2017. The study may be forwarded to the IRB for review.

Mario Eisenberger, MD, Chair
SKCCC Clinical Research Review Committee
